# Angular changes in implants placed in the anterior maxillae of adults: a cephalometric pilot study

**DOI:** 10.1007/s00784-020-03445-8

**Published:** 2020-07-13

**Authors:** Balazs Feher, Reinhard Gruber, Andre Gahleitner, Ales Celar, Philipp Luciano Necsea, Christian Ulm, Ulrike Kuchler

**Affiliations:** 1 Department of Oral Biology, University Clinic of Dentistry, Medical University of Vienna, Sensengasse 2a, 1090 Vienna, Austria; 2 Department of Oral Surgery, University Clinic of Dentistry, Medical University of Vienna, Vienna, Austria; 3 Department of Periodontology, School of Dental Medicine, University of Bern, Bern, Switzerland; 4 Department of Radiology, University Clinic of Dentistry, Medical University of Vienna, Vienna, Austria; 5 Department of Orthodontics, University Clinic of Dentistry, Medical University of Vienna, Vienna, Austria; 6 Department of Dental Training, University Clinic of Dentistry, Medical University of Vienna, Vienna, Austria

**Keywords:** Growth, Implant, Cephalometry, Pilot study

## Abstract

**Objectives:**

Completion of adolescent growth represents the earliest time point for implant placement, yet craniofacial growth persists into adulthood and may affect implant position. We aimed to assess whether implants placed in the anterior maxillae of adults show angular changes over time.

**Material and methods:**

We conducted a cephalometric pilot study in postpubertal patients with no growth disorders, skeletal malformations, or parafunctions. The patients received a single implant in the anterior maxilla and no orthodontic or orthognathic treatment afterwards. We measured angular changes of implants and central incisors on cephalograms taken immediately and after at least 5 years postoperatively with the Sella-Nasion line (SNL) and the nasal line (NL) as references. Changes in implant-SNL angles were the primary outcome.

**Results:**

In 21 patients (30.2 ± 11.5 years at surgery) after a mean follow-up of 8.6 ± 1.3 years, implant-SNL angles and implant-NL angles changed in 81% and 57% of implants, respectively. Implant-SNL changes ranged from 3° counterclockwise to 4° clockwise and were more prevalent in males (100% vs. 58%) and patients under 30 at surgery (85% vs. 63%); mean absolute differences were larger in males (1.8 ± 1.0° vs. 1.3 ± 1.4°) and patients under 30 at surgery (1.5 ± 1.4° vs. 1.1 ± 1.4°). Incisor-SNL angles and incisor-NL angles changed in 89% and 32% of incisors, respectively.

**Conclusions:**

Implants placed in the anterior maxillae of adults show modest angular changes over time.

**Clinical relevance:**

Changes in implant angles have potential functional and esthetic consequences.

**Electronic supplementary material:**

The online version of this article (10.1007/s00784-020-03445-8) contains supplementary material, which is available to authorized users.

## Introduction

Osseointegration is a “functional ankylosis” similar to that observed in teeth following injuries [1, 2]. Dental implants, like ankylosed teeth, do not follow the growth of the alveolar processes during eruption [3]. The placement of implants in growing jawbones thus results in the submersion of the implants over time, relative to adjacent erupting teeth [4]. In order to avoid this phenomenon, the earliest time point recommended for implant placement is following the end of adolescence, when growth is thought to be completed [5, 6]. However, craniofacial growth persists into adulthood [6, 7], causing significant dimensional changes of the facial skeleton in the long term [8–10]. With regard to dental implants, the clinical implications of this residual growth have largely been underestimated [11, 12]. Previous work has suggested vertical changes in implants as a result of continuous growth in adults [13, 14]. Recent data have given support to these findings and even pointed to a potentially high prevalence of infraocclusion [15–18]. Observed in adults, the described changes could not be explained by adolescent growth.

Humans are among the few species in which an adolescent growth spurt can be observed [19]. This period of significant increase in height and weight [20] triggers major changes in the jawbones [21]. Implant therapy during adolescence is restricted to cases of extensive hypodontia [22]. Adolescence ends with the closing of the epiphyses of long bones, typically around 18 years of age in males and 15 in females [23]. Some surgical protocols consider individual variability in aging and thus recommend a more conservative approach of placing implants at a slightly higher age [24]. With most of the skeletal growth completed by the end of puberty, implant placement starting at early adulthood is generally considered safe. Nevertheless, findings on the effects of continuous craniofacial growth have raised the question whether clinically relevant changes still can occur in the adult patient.

Compared with previous work describing vertical changes [13–18], data on possible angular changes in implants due to residual craniofacial growth in adulthood are lacking. Understanding potential angular changes in implants is important for multiple reasons. In the anterior maxilla, the palatal crown surfaces of incisors guide protrusion and canines play an important role in guiding laterotrusion [25]; changes in implant angles could lead to functional issues. Moreover, implant crown esthetics play a role in achieving clinical success [26]. It is apparent that in an exposed area such as the anterior maxilla, angular changes could undermine optimal results. For these reasons, the assessment of potential angular changes in implants is also of high clinical relevance. Cephalometry is a routine radiographic tool used in orthodontics and orthognathic surgery. Structures of the head skeleton as well as their spatial relationships are routinely measured using cephalometry [27]. To understand the possible effect of residual craniofacial growth on implant angles, we applied cephalometry in this pilot study to measure long-term angular changes in implants in the anterior maxillae of adult patients. To put potential angular changes in implants in perspective, we further measured long-term changes in the angles of maxillary central incisors.

## Materials and methods

### Experimental design

We conducted a long-term cephalometric pilot study that was designed in accordance with the Declaration of Helsinki. This study was conducted at a single center, the Medical University of Vienna, University Clinic of Dentistry. The study protocol was approved by the ethics committee of the Medical University of Vienna (*No.* 2174/2018). All recruited patients were fully informed about the procedure, the materials to be used in this study, their estimated exposure to radiation, the benefits, and potential risks and complications stemming from their participation in this study. All patients gave their written consent prior to participation in this study.

### Inclusion and exclusion criteria

We included patients that (i) received a single implant in the anterior maxilla (i.e., canine to canine), (ii) were at least 18 years of age at surgery, and (iii) received their implants at least 5 years prior to this study. We excluded patients with (i) birth defects with or without skeletal malformations (e.g., cleidocranial dysplasia), (ii) congenital growth disorders (e.g.*,* congenital growth hormone deficiency), (iii) parafunctions (e.g.*,* tongue thrust), (iv) traumatic injuries to the region of interest prior to or following implant therapy, (v) complications relating to the implant (e.g., peri-implantitis, fracture), as well as vi) orthodontic therapy, or (vii) orthognathic surgery (e.g., Le Fort osteotomy) following implant placement.

### Cephalometry

**Fig. 1:**
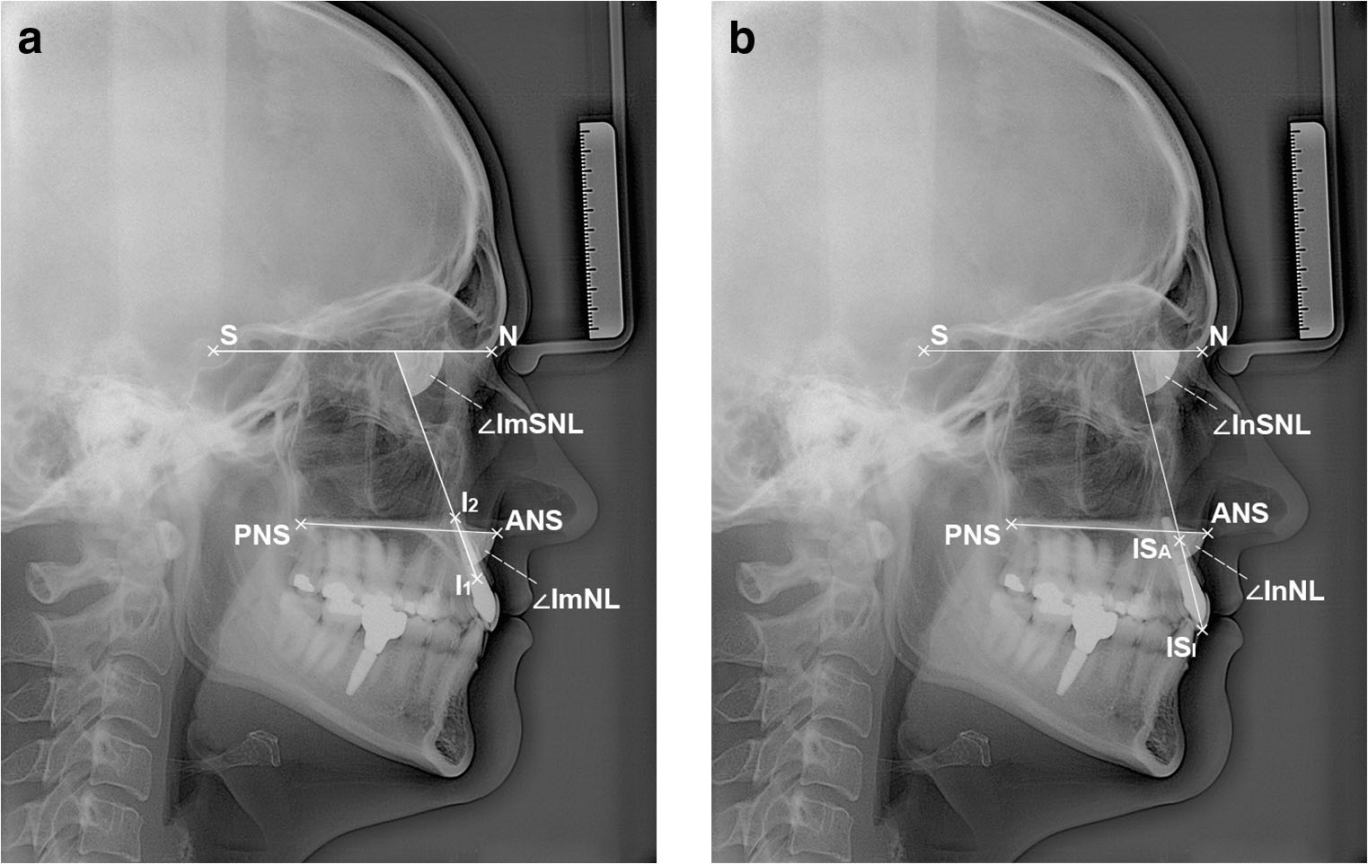
Radiographic parameters. **A** Implant angles. ANS anterior nasal spine, I1 implant shoulder, I2 implant apex, N Nasion, PNS posterior nasal spine, S Sella, ∠ImNL implant-NL angle, ∠ImSNL implant-SNL angle. **B** Incisor angles. ANS anterior nasal spine, ISA incision superius apicale, ISI incision superius incisale, N Nasion, PNS posterior nasal spine, S Sella, ∠InNL incisor-NL angle, ∠InSNL incisor-SNL angle

We used postoperative lateral cephalograms as baseline and took one follow-up lateral cephalogram per patient. Both cephalograms were taken in the same setting and using the same parameters (75 kV, 32 mAs, 3.9 m source-to-mid-sagittal-plane distance). The cephalograms were precisely standardized prior to analysis using a raster graphics editor (Photoshop, Adobe, Mountain View, CA, USA). Facial growth type was determined using Björk’s sum of the saddle angle, articular angle, and gonial angle [28]. The implant axis was defined as the straight line connecting the implant shoulder to the implant apex. The long axis of the maxillary central incisor (incisor axis) was defined as the straight line connecting the incision superius incisale to the incision superius apicale. We assessed these axes in relation to two reference structures. The Sella-Nasion line (SNL) was designated as the primary reference structure due to its stability [29]. The nasal line (NL) connecting the anterior nasal spine to the posterior nasal spine was designated as the secondary reference structure. Implant-SNL, implant-NL, incisor-SNL, and incisor-NL angles were then measured in anterior direction (Figs. 1a–b). The primary outcome of this study was any change in implant-SNL angles; secondary outcomes were changes in implant-NL, incisor-SNL, and incisor-NL angles. All lateral cephalograms were evaluated by a single researcher (PLN), and all measurements were evaluated by two different researchers (BF, UK) before going into analysis. To further ensure accuracy, the measuring researcher (PLN) was calibrated by unknowingly evaluating 21% of the complete radiographic dataset twice. The duplicate measurements were then compared by a different researcher (BF). Based on the comparison of the duplicates, the intraclass correlation coefficient (ICC) of the evaluator was 99% (deviation range 0 to 1°).

### Statistics

Consistent with the pilot nature of this study, no sample size was calculated prior to patient enrollment, and statistical analyses were descriptive in nature. Data were first collected in a spreadsheet (Excel 16.29.1 for Mac, Microsoft Corporation, Redmond, WA, USA), checked for possible errors, and consequently analyzed using the R statistical computing environment (Version 3.6.1, R Core Team, Vienna, Austria). Descriptive statistical methods were used for subject characteristics and basic comparisons of subgroups (e.g., sex and age distribution). Mean values and standard deviations (SDs) were calculated for numerical variables. Kernel density estimates and histograms were used to visualize numerical variables (Supplementary Figures 1a–c). Linear regression analysis was further used to assess changes in the implant angles between baseline and follow-up. The implant angle at follow-up was set as the dependent variable, with the implant angle at baseline, sex, age, Björk’s angle sum, facial growth type, and follow-up time serving as independent variables.

## Results

### Study population

A total of 21 patients (mean age at follow-up: 38.9 ± 11.2 years, age range 26–58 years, 57% female) completed the study after a mean follow-up time of 8.6 ± 1.3 years (range 6.6–10.9 years). The patients’ mean age at surgery was 30.2 ± 11.5 years (range 18–52 years). With regard to facial growth type, 67% of patients was brachyfacial (mean Björk’s sum 385.3 ± 4.2°, range 376–390°), 24% was mesofacial (mean Björk’s sum 395.0 ± 2.1°, range 392–397°), and 10% was dolichofacial (401.5 ± 0.7°, range 401–402°). Subject characteristics are presented in Table 1 and Supplementary Figures 1a–c.

**Table 1:** Subject characteristics

Total study population, *n*	21
Sex, *n* (%)	
Females	12 (57)
Males	9 (43)
Age, mean ± SD (range) in years	
At surgery	30.2 ± 11.5 (18–52)
At follow-up	38.9 ± 11.2 (26–58)
Growth type, *n* (%)	
Brachyfacial	14 (67)
Mesofacial	5 (24)
Dolichofacial	2 (10)
Follow-up, mean ± SD (range) in years	8.6 ± 1.3 (6.6–10.9)

*SD* standard deviation

### Changes in implant angles

To investigate angular changes in implants, we compared implant-SNL and implant-NL angles between baseline and follow-up radiographs. With regard to implant-SNL angles, changes were found in 81% of implants. A counterclockwise rotation (− ^*ve*^ angular change) was found in 62% of implants (mean ± SD − 1.8 ± 1.0°, range − 1 to − 3°). A clockwise rotation (+ ^*ve*^ angular change) was found in 19% of implants (mean ± SD 2.4 ± 1.1°, range 1 to 4°). No changes in implant-SNL angles were found in 19% of implants. Changes in implant-SNL angles were more prevalent in males than in females (100% vs. 58%). Mean absolute differences between baseline and follow-up were also larger in males than in females (1.8 ± 1.0° vs. 1.3 ± 1.3°). Changes in implant-SNL angles were more prevalent in patients under 30 at surgery than patients at least 30 years old at baseline (85% vs. 63%). Mean absolute differences between baseline and follow-up were slightly larger between patients under 30 and patients at least 30 years old at surgery (1.5 ± 1.4° vs. 1.1 ± 1.4°).

With regard to implant-NL angles, changes were found in 57% of implants. A counterclockwise rotation (− ^*ve*^ angular change) was found 29% of implants (mean ± SD − 1.7 ± 1.2°, range − 4 to − 1°). A clockwise rotation (+ ^*ve*^ angular change) was found in 29% of implants (mean ± SD 1.5 ± 0.4°, range 1 to 3°). No changes in implant-NL angles were found in 43% of implants. Changes in implant-NL angles were more prevalent in males than females (78% vs. 42%). Mean absolute differences between baseline and follow-up were slightly larger in males than in females (1.0 ± 0.7° vs. 0.8 ± 1.3°). Changes in implant-NL angles were slightly more prevalent in patients at least 30 years old at baseline than patients under 30 at surgery (63% vs. 54%). Mean absolute differences between baseline and follow-up did not vary between patients under 30 and patients at least 30 years old at surgery (0.9 ± 1.3° vs. 0.9 ± 0.8°). A summary of changes in implant angles is presented in Table 2.

**Table 2:** Changes in implant angles

	Implant-SNL angle				Implant-NL angle			
	Prevalence (%)	Range (deg.)	Mean ± SD (deg.)	Abs. mean ± SD (deg.)	Prevalence (%)	Range (deg.)	Mean ± SD (deg.)	Abs. mean ± SD (deg.)
Sex								
Female	67	− 4 to 3	− 0.5 ± 1.8	1.3 ± 1.3	42	− 4 to 3	0.0 ± 1.6	0.8 ± 1.3
Male	100	− 3 to 3	− 0.9 ± 1.9	1.8 ± 1.0	78	− 2 to 2	− 0.1 ± 1.3	1.0 ± 0.7
Age groups								
< 20 at surgery	100	− 2 to 3	0.2 ± 2.2	1.8 ± 0.9	60	− 1 to 3	0.6 ± 1.5	1.0 ± 1.2
20–29 at surgery	63	− 4 to 3	− 0.2 ± 2.0	1.2 ± 1.5	50	− 4 to 1	− 0.1 ± 1.6	0.9 ± 1.4
30–39 at surgery	100	− 1.5 to − 1	− 1.3 ± 0.4	1.3 ± 0.4	50	− 1 to 0	− 0.5 ± 0.7	0.5 ± 0.7
≥ 40 at surgery	83	− 3 to 0	− 1.8 ± 1.2	1.8 ± 1.2	67	− 2 to 2	− 0.3 ± 1.4	1.0 ± 0.9
Growth type								
Brachyfacial	79	− 4 to 3	− 0.4 ± 2.0	1.6 ± 1.2	57	− 4 to 3	− 0.2 ± 1.5	0.9 ± 1.2
Mesofacial	80	− 3 to 0	− 1.7 ± 1.3	1.7 ± 1.3	60	− 2 to 2	0.2 ± 1.5	1.0 ± 1.0
Dolichofacial	100	− 0.5 to 1	0.3 ± 1.1	0.8 ± 0.4	50	0 to 1	0.5 ± 0.7	0.5 ± 0.7

*Abs.* absolute, *deg.* degree, *NL* nasal line, *SD* standard deviation, *SNL* Sella-Nasion line

### Changes in incisor angles

To put the changes in implant angles in perspective, we compared the incisor-SNL and incisor-NL angles between baseline and follow-up radiographs. Incisor angles could not be assessed in 2 patients due to missing incisors at follow-up. With regard to incisor-SNL angles, changes were found in 89% of incisors (range − 5 to 4°). Changes in incisor-SNL angles were slightly more prevalent in males than females (100% vs. 83%) as well as in patients at least 30 years old at baseline than patients under 30 at surgery (100% vs. 83%). With regard to incisor-NL angles, changes were found in 32% of incisors (range − 4 to 3°). Changes in incisor-NL angles were slightly more prevalent in females than males (42% vs. 33%) as well as in patients under 30 at surgery than patients at least 30 years old at baseline (33% vs. 29%). A summary of changes in incisor angles is presented in Table 3.

**Table 3:** Changes in incisor angles

	Incisor-SNL angle				Incisor-NL angle			
	Prevalence (%)	Range (deg.)	Mean ± SD (deg.)	Abs. mean ± SD (deg.)	Prevalence (%)	Range (deg.)	Mean ± SD (deg.)	Abs. mean ± SD (deg.)
Sex								
Female	83	− 5 to 4	− 0.1 ± 2.4	1.7 ± 1.6	42	− 4 to 3	0.0 ± 1.7	0.8 ± 1.5
Male	100	− 2 to 3	0.0 ± 1.8	1.6 ± 0.7	33	− 3 to 3	0.2 ± 1.6	0.9 ± 1.4
Age groups								
< 20 at surgery	100	− 5 to 1	− 0.8 ± 2.7	2.0 ± 1.7	20	− 4 to 0	− 0.8 ± 1.8	0.8 ± 1.8
20–29 at surgery	71	0 to 4	1.6 ± 1.5	1.6 ± 1.5	43	0 to 3	0.9 ± 1.2	0.9 ± 1.2
30–39 at surgery	100	− 1 to 1	0.0 ± 1.4	1.0 ± 0.0	0	–	–	–
≥ 40 at surgery	100	− 2 to − 1	− 1.6 ± 0.5	1.6 ± 0.5	40	− 3 to 3	0.0 ± 2.1	1.2 ± 1.6
Growth type								
Brachyfacial	92	− 2 to 3	0.3 ± 1.6	1.3 ± 0.8	8	0 to 1	0.1 ± 0.3	0.1 ± 0.3
Mesofacial	80	− 2 to 4	− 0.2 ± 2.5	1.8 ± 1.5	60	− 3 to 3	0.6 ± 2.5	1.8 ± 1.6
Dolichofacial	100	− 5 to 1	− 2.0 ± 4.2	3.0 ± 2.8	100	− 4 to 2	− 1.0 ± 4.2	3.0 ± 1.4

*Abs.* absolute, *deg.* degree, *NL* nasal line, *SD* standard deviation, *SNL* Sella-Nasion line

### Linear regression analysis

**Fig. 2:**
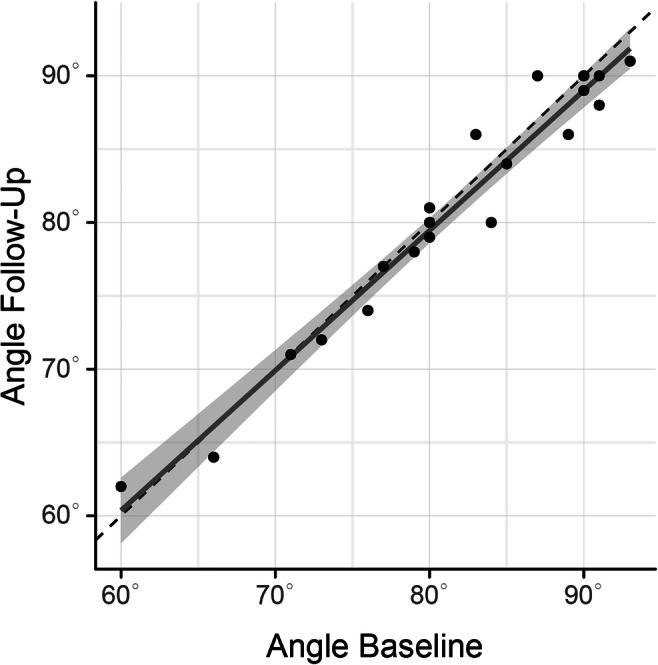
Linear regression analysis. The dashed line shows no change. The dark gray line represents the regression line. The light gray area surrounding the dark gray line represents the corresponding 95% confidence interval. Slope of regression = 0 (*p* < 0.001)

To further analyze changes in implant angles as well as determine whether demographic or growth-related factors could have an effect on them, we applied linear regression analysis to implant-SNL angles in an explorative manner. The analysis returned a slope of regression of 0 (*p* < 0.001) (Fig. 2). Further, none of the assessed predictors (implant angle at baseline, sex, age, Björk’s angle sum, facial growth type, and follow-up time) had an influence on the implant angle at follow-up.

## Discussion

Evidence on long-term changes in implant positions in adult patients is accumulating. Following early data from preclinical models [4, 30] and clinical studies in adolescents [31], findings from adults have substantiated the possibility of vertical changes in implant position, up to the point of infraocclusion [13–16]. The present pilot study is the first to report angular changes in implants over the course of adulthood. Based on a cephalometric analysis, we found that after a mean follow-up time of 8.6 years, 81% of implants placed in the anterior maxillae showed modest rotational changes ranging from 3° counterclockwise to 4° clockwise with reference to the SNL and 57% of implants showed rotational changes with reference to the NL. These findings are important for they show the possibility of angular changes in implants placed in adult patients. We further found that the prevalences, ranges, and means of implant-SNL and incisor-SNL angles showed similarities over time. Across both implants and natural incisors, SNL-referenced angles showed more prevalent and larger changes overall than NL-referenced angles. Taken together, these findings suggest that residual craniofacial growth could affect the maxilla as a whole and the angular changes cannot be explained solely by the continuous development of the alveolar process.

Our primary findings relate to those of others as at 81%, the prevalence of changes in implant-SNL angles in our patient cohort is comparable to that of vertical changes (73%) described recently [16]. In our patient cohort, changes in implant-SNL angles were found in 58% of females and 100% of males and changes in implant-NL angles were found in 42% of females and 78% of males. These sex differences are in contrast to the findings of others as with just one exception [32], previous work did not identify a predisposing role of sex on vertical changes in implants [13, 17, 18, 33]. With regard to age at surgery, changes in implant-SNL angles were found in 85% of patients under 30 and 63% of patients at least 30 years old at surgery; differences between these two age groups were smaller for changes in implant-NL angles, incisor-SNL angles, and incisor-NL angles. The differences between different age groups observed for changes in implant-SNL angles are in accordance with some previous findings [33] and in contrast to others [13, 16, 17]. The gradual decline of growth over time could explain how age at surgery influences long-term changes in implants. However, the existing literature does not unequivocally back up that theory [13, 16, 17]. We further found that the patient cohort showed a high individual variety in the extent and directions of rotational changes. Consistent with the pilot nature of this study, we did not conduct tests for statistical significance. In order to test the significance of angular changes or evaluate potential predictors, the threshold for a clinically relevant angular change has to be defined first by the scientific community. To assist future research into this area, we calculated sample sizes for theoretical thresholds of clinical relevance of 1 to 7° (Supplementary Table 1).

Computer-aided standardization prior to analysis, blinded observer calibration (ICC = 99%, deviation range 0 to 1°), and evaluating all measurements by the observer by two different researchers helped ensure precision and limit measurement error. Cephalometric analysis is inherently observer dependent. Intra-observer variability thus has to be minimized prior to analysis. Particular attention was given to the consistent marking of the Sella as it is a “floating” landmark; all other landmarks relevant to the analysis of angular changes are discrete structures (e.g.*,* nasofrontal suture, implant body, central maxillary incisor). We thus believe the angular changes measured are not due to measurement error. The changes in implant-SNL angles ranging from 3° counterclockwise to 4° clockwise are not so substantial as to prevent implant placement starting at early adulthood. Nevertheless, the data give support to previous work [16–18] highlighting the relevance of continuous craniofacial growth in implant dentistry. It remains open at what threshold angular changes become relevant to the patient; the present study did not evaluate that as the possibility of angular changes first had to be confirmed. While vertical changes are noticed by over 60% of affected patients, they are not necessarily dissatisfied as a result [16, 18]. Nevertheless, the esthetics of implant restorations in the anterior maxilla are highly relevant to patients. It is thus reasonable to assume that angular changes in the esthetic zone could cause a high degree of dissatisfaction.

Limitations of the present pilot study include its retrospective design, its relatively small sample size, and its reliance on two-dimensional radiographic imaging with the inherent limitations of lateral cephalography (e.g., double contours). Alternatives to lateral cephalometry include three-dimensional cone beam computer tomography. However, metal streak artifacts associated with computer tomography could make it difficult to accurately measure implant angles. Three-dimensional magnetic resonance cephalometry [34] could be utilized to overcome the potential limitations associated with the use of lateral cephalograms [35, 36]. The increasing amount of data supporting changes in implants placed in adults underscores the importance of future research into this field. Further studies with a prospective study design could take advantage of higher sample sizes and three-dimensional cephalometry to gain a more profound understanding of growth processes in adulthood and better evaluate angular implant changes in the anterior maxillae of adults. In the present study, regression analysis failed to identify significant explanatory factors for the observed changes in implant-SNL angles. Nevertheless, the findings should be considered relevant and basically favorable because while we showed that angular changes can occur in implants over the course of adulthood, their scale does not indicate that we should reconsider implant therapy starting at early adulthood.

## Conclusions

Within the limitations of this pilot study, it can be concluded that 81% of implants placed in the anterior maxillae of adult patients show angular changes in the long term, ranging between 3° counterclockwise and 4° clockwise with reference to the SNL. Our findings give support to previous work describing the effects of continuous craniofacial growth in implant dentistry.

## Electronic supplementary material

ESM 1 (DOCX 14 kb).

ESM 2 Histograms and kernel density estimates. **A** age in years. **B** follow-up time in years. **C** björk’s angle sum in degrees. (PNG 14913 KB).

High resolution image (TIF 3460 kb).
