# Supplementary material for: Angular changes in implants placed in the anterior maxillae of adults: a cephalometric pilot study
Source: Clin Oral Investig. 2020 Jul 13;25(3):1375–81. doi: 10.1007/s00784-020-03445-8 (PMC7878256; doi:10.1007/s00784-020-03445-8)
Supplement: Supplementary file 1 — (DOCX 14 kb). [file 784_2020_3445_MOESM1_ESM.docx]

## Supplementary Table 1 SAMPLE SIZE CALCULATION

| **Change in implant-SNL angle, deg.** | **Sample size, n Power = 0.80** | **Sample size, n Power = 0.90** | **Sample size, n Power = 0.95** | **Sample size, n Power = 0.99** |
| --- | --- | --- | --- | --- |
| 1 | 494 | 660 | 815 | 1152 |
| 2 | 125 | 167 | 206 | 290 |
| 3 | 57 | 75 | 93 | 130 |
| 4 | 33 | 44 | 53 | 74 |
| 5 | 22 | 29 | 35 | 48 |
| 6 | 16 | 21 | 25 | 34 |
| 7 | 13 | 16 | 19 | 26 |

Deg., degree; SNL, Sella-Nasion line.
