# Supplementary figures and images for: Angular changes in implants placed in the anterior maxillae of adults: a cephalometric pilot study
Source: Clin Oral Investig. 2020 Jul 13;25(3):1375–81. doi: 10.1007/s00784-020-03445-8 (PMC7878256; doi:10.1007/s00784-020-03445-8)

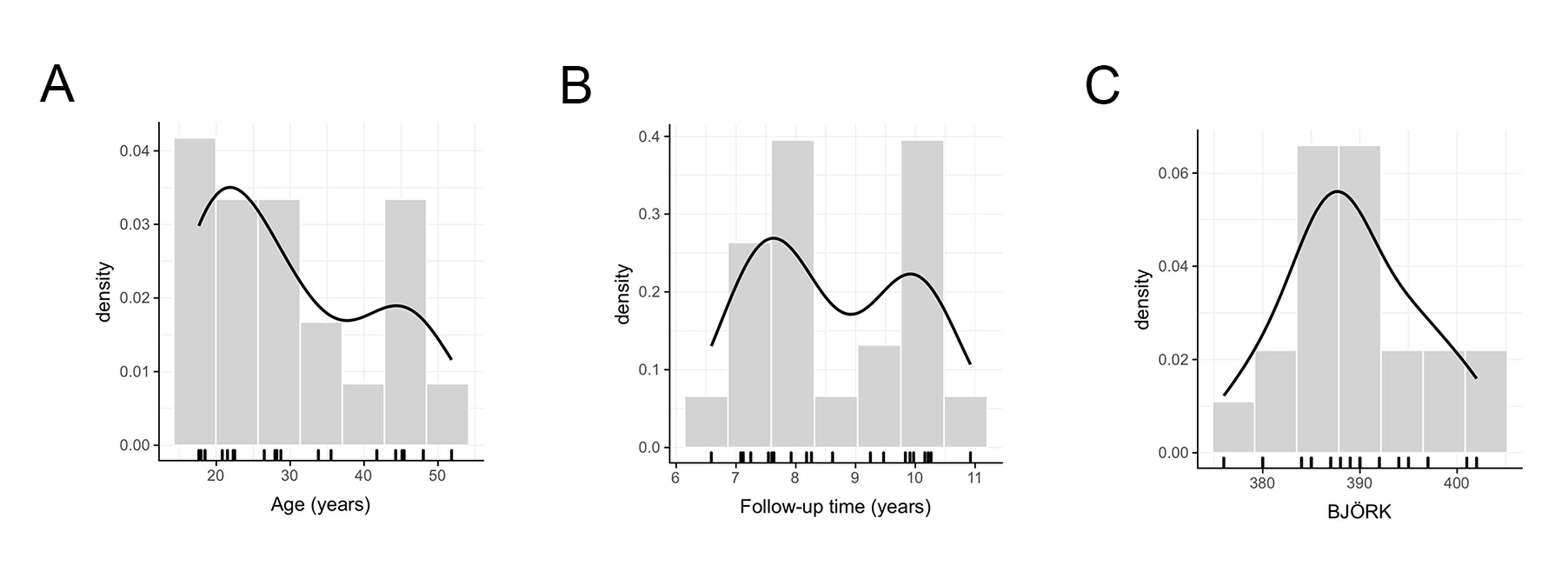

Supplement: Supplementary file 2 — Histograms and kernel density estimates. A age in years. B follow-up time in years. C björk’s angle sum in degrees. (PNG 14913 KB). [file 784_2020_3445_Fig3_ESM.png]

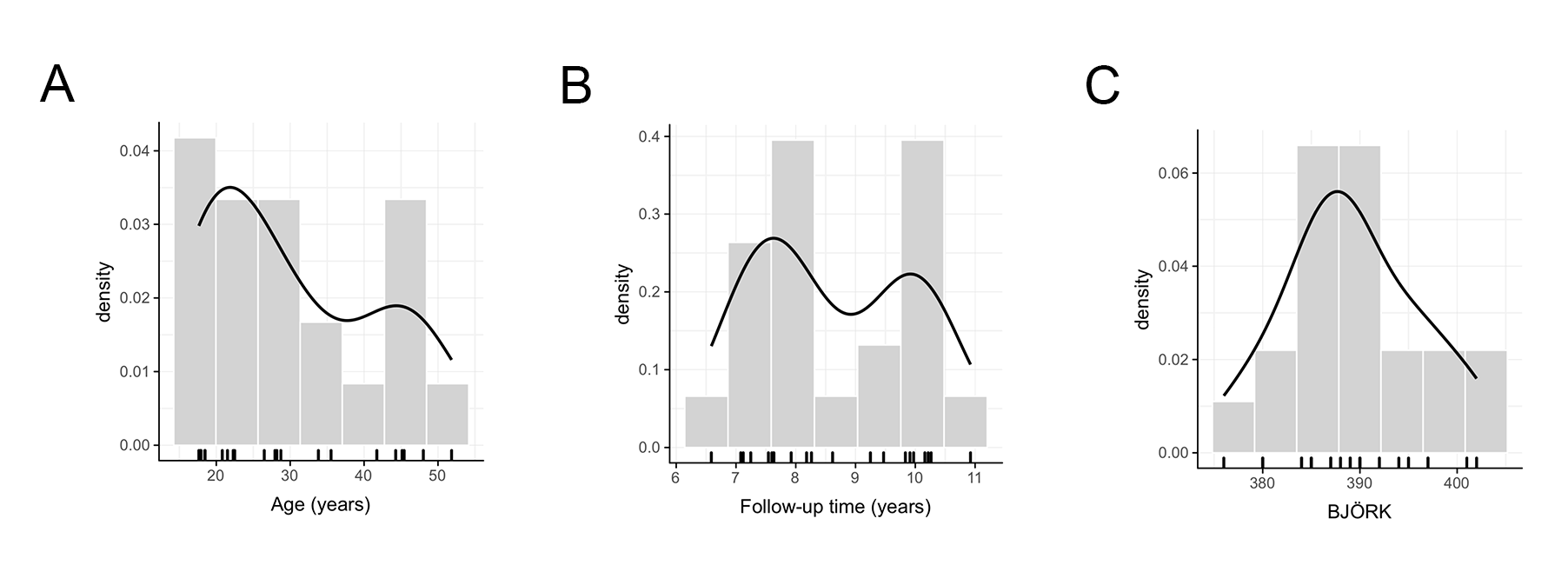

Supplement: Supplementary file 3 — High resolution image (TIF 3460 kb). [file 784_2020_3445_MOESM2_ESM.tif]
